# Optimizing Population Health: Strategies for Advanced Level Nurses

**DOI:** 10.5195/jmla.2025.2201

**Published:** 2025-10-23

**Authors:** Muhammad Taufan Umasugi

**Affiliations:** 1 umasugi53@gmail.com, STIKes Maluku Husada, Jl. Lintas Seram Kairatu, Seram bagian Barat. Indonesia

*Optimising Population Health: Strategies for Advanced Nurses* offers a down-to-earth, in-depth review of current population health content suitable for advanced nurses. Written for Master of Science in Nursing (MSN) students at the University of West Florida, the book aims to provide essential content for advanced nurses to study the most pressing issues in contemporary population health. Consisting of four sections—population health, cultural humility, health promotion, and contemporary health issues—this text presents evidence-based approaches, strategies, and resources designed to support nurses in developing practice and leadership skills in the definition and approach to population health. The primary audience is MSN students, but it is also a resource for practising nurses involved in leadership, education, or public health roles.

## Chapter 1: Introduction to Population Health

Population Health: Chapter one lays the foundation by introducing the reader to the basics of population health—one of the main components of advanced practice nursing skills. This chapter covers the demographic and geographic framework of the population, which provides a basis for understanding how population health outcomes are influenced by the broader social and environmental context. Each chapter ends with evidence-based recommendations that explain the role of health assessment in developing population health strategies and exploring the Social Determinants of Health (SDOH). Public health priorities in the United States are succinctly summarised in Healthy People 2030, one of the pillars of this chapter and a major strength of the book. This project sets the standard that advanced nurses in the field of public health must achieve. In this chapter, the emphasis on health partnerships and collaboration between sectors is very important, as it highlights the importance of an interdisciplinary approach in dealing with complex health issues. This section is particularly useful for MSN students who are studying for leadership positions where managing or leading such unions will be very important.

## Chapter 2: Cultural Humility

Unlike the traditional idea of cultural competence, where one assumes a broad understanding of the entire culture, cultural humility emphasises a lifelong learning journey, recognising the need for healthcare practitioners to remain open-minded and open to the different perspectives and experiences of the patients they treat. Cultural humility is a lifelong process of self-evaluation and self-criticism that is key to combating the implicit biases that impact patient care, this chapter explains. This chapter's emphasis on implicit bias, and its damaging impact, is particularly relevant as healthcare organisations seek to eliminate discriminatory differences in care. This chapter explains how awareness of Adverse Childhood Experiences (ACEs) and Trauma-Informed Care (TIC) will help advanced nurses identify and address the long-term impact of trauma on patient populations. Particularly relevant for nurses who practice in public health or care for vulnerable populations, where understanding and addressing trauma can form the basis of patient care. This chapter's in-depth presentation on cultural humility, implicit bias, and trauma should be read by all of us who work in patient care as well as those who try to advocate for health equity.

## Chapter 3: Health Promotion

This chapter links the concept of population health with a strategy of action to improve health. This chapter discusses the Levels of Prevention (primordial, primary, secondary, tertiary, and quaternary prevention) and relevant examples of these levels of prevention in practice. The discussion of health promotion theories, such as the Health Belief Model, Social Cognitive Theory, and the Transtheoretical Model, provides a basis for nurses to plan interventions that can lead to sustainable health and disease prevention. In a very helpful section, this chapter discusses health literacy, an aspect of population health planning that is rarely referred to. How health literacy correlates with health outcomes is important for nurses as they seek to educate, engage, and promote healthy communities. This chapter provides a practical approach to outlining health promotion strategies that are important for advanced nurses who are not only responsible for the specific health problems of their clients, but also responsible for offering preventive measures for future health problems.

## Chapter 4: Contemporary Health Issues

This chapter focuses on several crucial global health challenges today such as the SDGs, climate change, and disaster management. As the role of APNs has an important role in controlling mass health crises, these topics are becoming increasingly important for their practice. This chapter inspires nurses to think about health interventions in a global context by aligning population health priorities with the SDGs. The sections relating to disaster management and infectious diseases are of particular concern given the increasing expression of global health emergencies such as pandemics and natural disasters. This chapter also discusses preparing students to face these challenges from a population health perspective for advanced practice nurses in leadership and education roles. Nurses are also encouraged to advocate for health policies that address the upstream determinants of health equity including climate change and socioeconomic inequalities, with clear next steps to help nurses understand how to use policy as a lever for change.

## Advantages of This Book

Optimising Population Health provides a strong blend of academic rigour and real-world support, especially with the widespread adoption of open educational resources (OER) in this book, making it very accessible to students and practitioners. Therefore, this book complements traditional textbooks in the field of population health, which can be limited by high costs and static content, and presents dynamic and updated material that can be modified and consolidated at no cost to users. As a freely available publication, this book can be updated from time to time so that students and health workers can have the latest population health strategies and tools they can use. The book also provides real-time digital resources for the learning experience through videos and interactive links. The book also delves into cultural humility and the importance of traumainformed care, making it a must-read for nurses serious about health equity.

## Potential Readership

This book is designed specifically for MSN students, especially the FNP, Nurse Educator, and Nurse Leader programmes. Due to its comprehensive approach, this book is also useful for practising nurses who wish to broaden their knowledge base in the fields of population health, health promotion, and advocacy. This book is timely for advanced practice nurses in leadership, policy-making, or public health roles, as it provides theoretical knowledge and practical tools that enable readers to consider how to manage new health challenges. As a nursing educator, this book is a very helpful resource when looking for current material to include in your courses, especially when teaching population health, public health nursing, or health policy courses. This knowledge will also be useful for health systems working to optimise care models by implementing population health strategies and interprofessional collaboration.

## Conclusion

Optimising the Health of Populations is a well-considered text that provides a fundamental understanding for practitioners who want to improve and/or develop their practice in the field of population health. Quest shows that the combination of theory, evidence-based strategies, and practical tools is invaluable for advanced practice nurses, educators, and policy advocates. This book stands out in its emphasis on cultural humility, implicit bias, and health promotion strategies, with the ultimate goal of advancing health equity and addressing global health challenges. The open access model of this book makes another major contribution, as access to the text allows it to be shared and adapted for various uses in nursing programmes at universities around the world. With its focus on developing nurses to lead in improving healthcare outcomes for those who are most vulnerable, this book will remain a real-time resource as the health of populations continues to evolve in various communities.

